# Hand-foot synkinesis in a patient with phenytoin intoxication

**DOI:** 10.1016/j.prdoa.2024.100237

**Published:** 2024-01-26

**Authors:** Gohei Yamada, Takanari Toyoda, Eiichi Katada, Noriyuki Matsukawa

**Affiliations:** a Department of Neurology, Nagoya City University West Medical Center, Aichi, Japan; b Department of Neurology, Nagoya City University Graduate School of Medical Sciences, Aichi, Japan

**Keywords:** Hand-foot synkinesis, Synkinesis, Phenytoin intoxication, Cerebellum

## Abstract

Here we report the first case of phenytoin intoxication that was closely associated with hand-foot synkinesis. This case suggests a close association between cerebellar dysfunction and hand-foot synkinesis. In patients with hand-foot synkinesis, lesions of not only the secondary motor areas but also the cerebellum should be considered.

## Highlights

• A case of phenytoin intoxication that was associated with hand-foot synkinesis.
• The close association between cerebellar dysfunction and hand-foot synkinesis.
• In hand-foot synkinesis, lesions of the cerebellum should be considered.

## 1 Introduction

Hand-foot synkinesis is an involuntary movement of the foot induced by the movement of the unilateral hand, or vice versa [1], [2]. This neurological sign is seen in patients with Parkinson’s disease (PD), essential tremor, Creutzfeldt-Jakob disease (CJD), and corticobasal syndrome [2], [3], [4]. Herein, we report the first case of phenytoin intoxication that led to hand-foot synkinesis.

## 2 Case Report

**Fig. 1:**
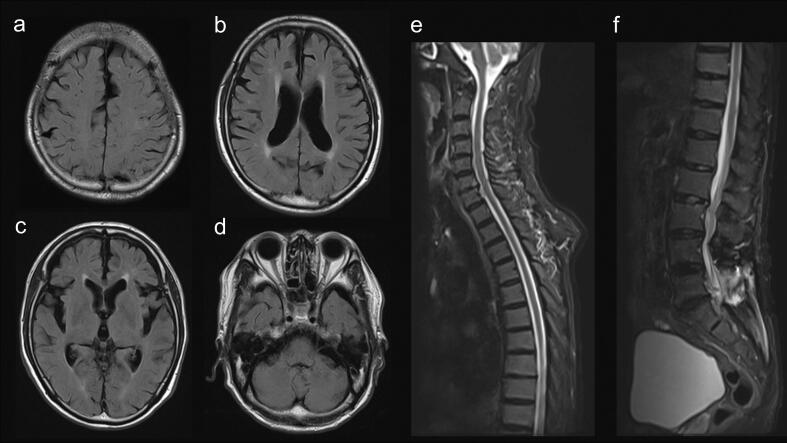
Brain and spine MRI. FLAIR MRI of the brain and T2WI MRI of the spine. Brain and spinal cord lesions are not shown (a-f). Mild stenosis in L2-4 and post-surgical signal changes in L5-S1 are shown (e, f).

An 82-year-old male patient developed epilepsy at the age of 12. Epilepsy was well controlled with phenytoin (200 mg/day) for 9 years. One year prior, the patient had undergone surgical procedures for lumbar canal stenosis. The patient acutely became unable to walk because of falling backward while standing. Five days later, the patient was transferred to our hospital via ambulance. Neurological examination revealed gaze-evoked horizontal nystagmus, mild dysarthria, retropulsion, dysmetria, and decomposition of the upper and lower extremities, which reflected cerebellar dysfunction (Video 1). The left tibialis anterior muscle was weak (MRC scale 4), and sensory impairment was present in the left calf and foot due to lumbar canal stenosis. Notably, involuntary foot movement was simultaneously observed when the patient repeated the extension-flexion movement of the fist and the pronation-supination movement of the hand (Video 2). Involuntary foot movements were induced by unilateral or bilateral hand movements and were predominantly observed on the right side. The rhythm, direction, and speed of the involuntary foot movement were similar to those of the right hand movement. While moving the left hand, the rhythm, direction, and speed of the involuntary foot movement were not necessarily similar to those of the left hand movement. These hand movement-induced foot movements involved the activation of non-mirroring muscles, which was compatible with hand-foot synkinesis. An extensive dystonic posture of the left fifth digit appeared during repeated extension-flexion movements of the right fist. The dystonic posture of the right foot, which consisted of inversion of the foot and extension of the great toe, appeared during repeated pronation-supination movements of the right hand. Mirror movement was observed in the left digits and left forearm during repeated extension-flexion movements of the right fist and pronation-supination movements of the right hand. Magnetic resonance imaging (MRI) of the brain and spinal cord revealed no causative lesions (Fig. 1). Serum phenytoin was 46.7 µg/ml. The patient was diagnosed with phenytoin intoxication. Phenytoin (200 mg/day) was promptly discontinued. On day 4, the serum concentration of phenytoin was 22.5 µg/ml. On day 6, gaze-evoked nystagmus, retropulsion, and gait impairment disappeared. The hand-foot synkinesis was almost completely resolved (Video 3). Thus, the clinical history suggested an association between cerebellar dysfunction and hand-foot synkinesis. Mild mirror movement in the left forearm was persistent. On day 8, the patient’s serum phenytoin was 6.7 µg/ml. Phenytoin was reinitiated at a dose of 100 mg/day. The patient was discharged from the hospital on day 12. Although the detailed cause of phenytoin intoxication remained unclear, the metabolism of phenytoin might decrease during the aging process.

## 3 Discussion

Although cerebellar dysfunction is common in phenytoin intoxication, hand-foot synkinesis has not been reported as a clinical manifestation of this condition. The contribution of secondary motor areas, including the SMA, dorsal and ventral premotor cortex, and cingulate gyrus to hand-foot synkinesis has been suggested in several case reports and a functional MRI study [2], [3], [4]. The hand and foot areas are separated in the primary motor cortex but overlap in the secondary motor areas. Dysfunction of the secondary motor areas could explain the simultaneous movement of the hand and foot. A potential association between cerebellar dysfunction and hand-foot synkinesis has not yet been reported. However, in the case of CJD with hand-foot synkinesis, the patient presented with cerebellar signs such as dysmetria on the finger-to-nose test, wide-based ataxic gait, and retropulsion during standing [2]. Moreover, fluorodeoxyglucose-positron emission tomography showed hypometabolism not only in the SMA and premotor cortex but also in the right cerebellum. In another report, functional MRI data of a patient with essential tremor and hand-foot synkinesis revealed activation not only in the SMA and foot motor cortex but also in a small area of the cerebellum [4]. The cerebellum is involved in surround inhibition and is related to motor overflow [5]. Therefore, the cerebellocortical pathway may be impaired in cases of hand-foot synkinesis, which could allow aberrant cerebellar output and induce hand-foot synkinesis.

In this case, mirror movement persisted even after the disappearance of cerebellar ataxia. The potential cause of mirror movement is dysfunction of the dorsal premotor cortex or reduction of interhemispheric inhibition, both of which play a role in suppressing contralateral movements. Hand-foot synkinesis may have been attributable to pre-existing dysfunction of the dorsal premotor cortex or reduction of interhemispheric inhibition in combination with cerebellar dysfunction.

In conclusion, we demonstrated a case of hand-foot synkinesis that was closely associated with phenytoin intoxication. Cerebellar dysfunction represents the potential mechanisms underlying hand-foot synkinesis.

## CRediT authorship contribution statement

**Gohei Yamada:** Conceptualization, Investigation, Writing – original draft. **Takanari Toyoda:** Investigation. **Eiichi Katada:** Investigation. **Noriyuki Matsukawa:** Supervision.

## Declaration of competing interest

The authors declare that they have no known competing financial interests or personal relationships that could have appeared to influence the work reported in this paper.
